# The exploitation argument against artificial companionship

**DOI:** 10.1007/s43681-026-01064-9

**Published:** 2026-06-30

**Authors:** Jonathon VandenHombergh

**Affiliations:** Department of Bioethics, National Institutes of Health, Bethesda, USA

**Keywords:** Artificial companions, Exploitation, Loneliness, Deception, Carebots

## Abstract

There is increasing interest in the use of artificial intelligence for companionship. Most resistance to this proposal appeals to the risk of deception: the idea that, because they lack the relevant mental states, artificial companions cannot authentically befriend their users. In this paper, I argue that it would be morally impermissible to befriend artificial companions *even if* there was no deception involved in doing so. This is because, without deception, such befriending would become *exploitative*: it would require users to take unjust advantage of certain vulnerabilities required for authentic friendship. I make the premises of this argument explicit and defend each in turn, both positively and from various objections. The resulting view is one on which—barring some interesting alternatives raised by those objections—non-deceptive artificial companionship is analogous to knowing participation in an arranged marriage.

## Introduction

An *artificial companion* is an artificial intelligence that acts like a friend.^1^ Examples include the chatbots Replika and Character.ai. After downloading and customizing either chatbot, you can converse with them through text or voice. More generally, you can *befriend* these and other artificial companions, and they can befriend you. I mean the term neutrally. “Befriending” is just behaving in ways common for human friendships, such as chatting or playing. It is not necessarily anything deeper than that. Still, many people report that such befriending is quite realistic. Some studies even suggest that it might improve your well-being [14, 29, 91]. It’s no surprise, then, that scholars are now considering the use of artificial companions for various social problems [36, 46]. The most obvious of these problems is the “epidemic of loneliness” [60]; cf. [32, 63, 102]. For whereas human companions are sometimes hard to come by, artificial ones are always there for you. Perhaps our epidemic of loneliness will soon be a thing of the past.

But there are critics. Most of them point out that artificial companions don’t *really* care about their users. The former lack emotions, intentions, and other features which human companions possess [55, 59, 88, 96]. In that case, befriending them seems to require deceit [38, 51, 58, 89] cf. discussion in [100]. Perhaps the artificial companion deceives the user into thinking that its friendship is genuine. Or perhaps the designers are to blame for the deceit—or the user herself (or even no one, as in [106]). In any case, the thought is that such deceit is wrong (though see [22]). This suggests that you shouldn’t befriend artificial companions, even if they might make you less lonely.

Yet this deceit is shaky. On the one hand, it’s not unreasonable to think that today’s artificial companions *do* care in the sense above. For all we know, they might just do so through unfamiliar algorithmic means [24]; cf. [12, 104]. On the more likely hand, even if these companions don’t care *now*, they might one day do so [9, 69, 83, 93]. Critics tend not to address these possibilities. That’s fair enough. After all, they raise deep philosophical challenges which can’t be solved in brief policy papers. But this doesn’t defeat the point. Critics *do* rest their case on a premise which could be(come) false.

Still, I think that they have the right idea. To strengthen their case, I will argue here that you shouldn’t befriend artificial companions *even if they did care about you*. The reason you shouldn’t is that doing so requires you to *exploit* them. Let’s call an artificial companion a “carebot” when it actually cares about us (and a “carefreebot” when it doesn’t).^2^ We can then give the argument step-by-step:

 (The Exploitation Argument)

1. A carebot wants to befriend you.
2. If a carebot wants to befriend you, then we made it that way.
3. If we made a carebot want to befriend you, then it’s exploitative for you to befriend it.
4. If it’s exploitative for you to befriend a carebot, then you shouldn’t befriend it.
5. So, you shouldn’t befriend a carebot.

If the premises of this argument (lines 1–4) are true, then its conclusion (line 5) must be true as well. I think that its premises *are* true. And I’ll defend that claim below, one premise per section. Along the way, I’ll address some minor objections. I’ll also define some unclear terms—“we,” “exploit,” and so on.

The term “care,” however, is just too complex for me to define here (cf. [80]).^3^ Yet no such definition is needed. The point of the exploitation argument is to show that you shouldn’t befriend an artificial companion even if it *doesn’t* deceive you. So when I say that a carebot cares, I really just mean that it has whatever features are needed for it to befriend you without deception. These features might be quite rich. A carebot might say things like “I love you” or “I want to see you” or “I’ve been thinking of you.” For this sort of befriending to avoid deception, each claim needs to be true. And that means the carebot is capable of love, desire, thought, and more. But I won’t even need all these features to make my point. I’ll argue for the features I *do* need, when relevant.

I should also be upfront about the word “exploitation.” As I mean it here, you exploit someone when you take unjust advantage of them. I’ll elaborate more on this definition later. But those familiar with the large philosophical literature on the topic might be skeptical of it (e.g., [101]). Among other things, it seems to rule out the possibility of fair exploitation. Yet most would agree that there is a *sense* of “exploitation” in which it just can’t be fair. That is the sense I have in mind here. If you insist that this sense is misguided, however, you can just replace “exploitation” with the clunkier phrase “unjust advantage-taking.” That’s ultimately what the exploitation argument is getting at anyway.

It's worth mentioning some related arguments, and why mine is different. Probably the most similar argument comes from a 2019 paper by Bartek Chomanski [13]. In that paper, Chomanksi argues that we’d have to be manipulative to make artificial servants as smart as human beings. Manipulation is quite similar to exploitation. And, like my argument, Chomanksi says that the problem can arise even if society benefits and the artificial beings keep their autonomy (cf. [72, 73]). However, carebots raise unique concerns, related to befriending without deception. And these concerns have to do with wronging the carebot itself, not (as Chomanski sees it [62]) with having a bad attitude. Eric Schwitzgebel (ms), sometimes with Mara Garza [82, 83], has also argued on behalf of artificial beings (see also [34]). This includes the idea that artificial beings should be free to explore values other than the ones we care about. I’ll argue later on that the exploitation of carebots also has to do with freedom. But my concerns about freedom will, once again, be unique to carebots who befriend without deception. I’ll discuss other similar arguments, usually in the footnotes, when relevant.^4^

A final caveat. The exploitation argument, like any argument, is limited. I’ll never be able to justify every assumption used in support of its premises. I *will* try to make these assumptions plausible. But some will end up being controversial no matter what I try (even after some detailed footnotes). In these cases, I hope at least that the assumptions will not be *im*plausible. At the same time, even controversial assumptions can be useful. Rejecting them means that the conclusion of the exploitation argument will need caveats. It will have to say that you shouldn’t befriend a carebot *unless* such and such assumptions are false. I find conclusions like this interesting in their own right. However, to keep things simple, I defend the argument directly and without mentioning these caveats. The reader is invited to add them as they wish.

## A defense of premise 1

Premise 1 says that a carebot wants to befriend you. This means that, unlike a carefreebot, a carebot does not *merely* befriend you. It also has a desire or preference to do so (even if it doesn’t seek you out on its own).^5^ The defense of this premise is simple, but not trivial. Suppose that a carebot does not want to befriend you. This might mean one of two things. First, it might mean that a carebot wants not to befriend you. Second, it might mean that a carebot neither wants to befriend you nor wants not to befriend you. In the first case, the carebot seems hostile. In the second case, the carebot seems indifferent. In both cases, therefore, the carebot does not seem to care about you (that is, it does not seem to have the features needed for befriending you without deception). But a carebot cares about you by definition. This means that a carebot must want to befriend you after all. To assume otherwise leads to a contradiction: a carebot that doesn’t care about you. In other words, premise 1 is true.

One might object that a carebot still *befriends* you in the two cases above. If that is true, then maybe it also *cares* about you in both cases. But recall that I am using “befriend” neutrally, to pick out behaviors like chatting. If befriending you is enough for care, then an angry or apathetic coworker cares about you whenever they play nice at work. That’s obviously not the case. All sorts of things can act in ways which seem caring without actually caring. So, this objection fails.

Another objection says that animals are indifferent in the sense above. In that case, perhaps a caring pet could befriend its owner, even though it neither wants to do so nor wants not to do so. If a caring pet is enough like a carebot, then the latter might also befriend you with indifference. One reply is that animals probably aren’t indifferent. True, their preferences might be less sophisticated than ours. But this does not mean that they lack preferences altogether [1, 43]; cf. [23]. Another reply is that a caring pet is not enough like a carebot. A carebot, after all, cares in a sophisticated way. It can express its care through conversation and other humanlike ways of befriending. A pet cannot do this. The sense in which a carebot cares, that is, suggests that it does not befriend you with indifference in the way that a pet might. Whichever reply we choose, the objection once again fails.

## A defense of premise 2

Premise 2 says that if a carebot wants to befriend you, then we made it that way. When I say “we,” I mean some group of human beings: designers, engineers, programmers, and maybe even users like you. When I say that we “made” a carebot someway or other, I mean that we caused it to be that way without using persuasion (or similar tactics). Training a large language model is a good example of this [7]. When the model fails in the task we are training it for, we do not try to convince it to do better. Instead, we tweak its weights until it succeeds. Customization is another good example. When you want a carebot to act like an older sibling, you don’t just ask it nicely. Rather, you go into the settings and choose the “older sibling” option (or what have you; cf. [77]).

With these terms in mind, premise 2 is hard to reject. Suppose first that we did not make a carebot want to befriend you. In this scenario, there are only two ways it might end up wanting to befriend you anyway. First, something (whether or not it’s us) might cause it to do so *with* persuasion. Second, something *other* than us might cause it to do so without persuasion—for short, “naturally.”^6^ Both options are open-ended. We can imagine many ways of persuading a carebot and many ways it might naturally want your friendship. The problem is that most of these ways are unlikely to succeed. In other words, given that we did not make a carebot want to befriend you, you’d have to be *lucky* for it to do so. This result is unacceptable. Even if you do not have to be very lucky, the entire point of carebots is to (more or less) guarantee friendship (cf. [13]). Without this guarantee, you might as well just try your luck with a human companion. Practically speaking, we should agree that if we did not make a carebot want to befriend you, then it won’t do so. And, logically speaking, this is the same as saying that if a carebot wants to befriend you, then we made it that way. In other words, we should agree with premise 2.

But perhaps we can imagine more likely paths to success. We might make a carebot with a cheery disposition, for example (though see [83]). You could then persuade this carebot through an ordinary chat, and it might be highly likely to want your friendship. Or perhaps we could create billions of different carebots for each user (though see [86]). It would then be highly likely that one of them—by sheer chance—wants to befriend you. Either case would give us something closer to a guarantee. And neither would require us to *make* a carebot want your friendship. Still, these possibilities are unrealistic. Among other things, they seem *harder* to accomplish than most ways of *making* a carebot want to befriend you. It’s probably easier to make a carebot want to befriend you than it is to persuade one, however cheerful it might be. And it’s definitely easier than making billions and hoping for the best.^7^ But even if we took one of these harder paths, we would *still* face something like the exploitation argument. That’s because it also seems exploitative to befriend a carebot readymade for your persuasion, or one among many mass-produced for your (accidental) benefit.^8^

A broader objection to premise 2 is related to the problem of free will [67]. The idea here is that we cannot *make* anything *want* something in the first place. For if one thing wants another, then (the thought goes) it does so freely. And if it does so freely (the thought continues), then it wasn’t caused to do so.^9^ But this implies that, if one thing wants another, then it wasn’t made to do so. The same reasoning applies just as well to carebots. So, premise 2 is false. Or, more carefully, either premise 1 or premise 2 is false—they can’t both be true.

There are multiple ways of addressing this objection. One is to deny that if something freely wants another thing, then it wasn’t caused to do so. To do this is to embrace *compatibilism* [52]. That is a popular solution to the problem of free will anyway. What seems to matter for freedom is not being uncaused, but being caused in the right way. If a random advertisement causes me to want coffee, then I probably don’t want it freely. But if my love of coffee causes me to want it, then I probably do. This is true (says the compatibilist) even if my love of coffee is itself caused by genetics or other forces outside my control. A similar story might hold for carebots. We might start by making a carebot which is curious about you. When it later wants to befriend you, it might do so because of its curiosity. In that case, its desire is caused. Yet it also seems free. That’s because its curiosity seems more like my love of coffee than a random ad. Unlike, say, direct customization, curiosity is the right kind of cause for free wanting. And this is true (says the compatibilist) even if that curiosity was caused by programming or other forces outside the carebot’s control.

Not everyone accepts compatibilism [98]. Luckily, the second response does not require it. Even if freedom is uncaused, we can still deny that wanting must be free. Consider a man and woman. Held at gunpoint, the man hands over his watch. The woman does similarly, but safely at the return counter. It seems that the woman wanted to hand over her watch, and that she did so freely. But now suppose that wanting must be free. In that case, the man either did *not* want to hand over his watch, or he did so *freely*. Neither option is plausible. He certainly wanted to hand over the watch, not least because he wanted to live ([103], p. 340). And if he did so freely, then his desire wasn’t too different from the woman’s. Yet it clearly was different. She, but not he, wanted freely. We cannot explain this difference unless we deny that wanting must be free.^10^

## A defense of premise 3

Premise 3 says that if we made a carebot want to befriend you, then it’s exploitative for you to befriend it. This premise is perhaps the most controversial. Start with the idea of exploitation. It’s “exploitative” for you to do something roughly when you take unjust advantage of someone by doing it [28, 101, 105].^11^ Different things can be taken advantage of, and different advantages can be taken. For example, a sweatshop owner takes advantage of his impoverished workers. The advantage taken is cheap labor. And since this advantage taking is clearly unjust, it’s exploitative for the owner to act as he does.

With this in mind, suppose that we made a carebot want to befriend you. Suppose also that you go on to befriend it. In that case, you are clearly taking some advantage of the carebot. Specifically, you are taking advantage of its desire to befriend you, and the advantage taken is the friendship itself. This advantage taking is perhaps morally okay. But if it is instead unjust, then you are also exploiting the carebot. And your advantage taking *does* seem unjust. For we have supposed that the desire you are taking advantage of is one which we made the carebot have. At the same time, we must have given the carebot an ability to want otherwise. Without this ability, its desire to befriend you seems meaningless. And that is inappropriate for a caring being. But now it looks like we gave the carebot an ability while causing that ability to be exercised only in one way: to want your friendship, to benefit you. We also did this without persuasion or similar tactics. In a word, we *rigged* the carebot from the start. That seems deeply unjust.^12^ If it is unjust, however, then it also seems unjust to take advantage of the desire we rigged.^13^ This shows that you are exploiting the carebot. More carefully, it shows that it’s exploitative for you to befriend the carebot given that we made it want to befriend you. And that is just what premise 3 claims.

An analogy might help. Consider a retelling of Shakespeare’s *Romeo and Juliet*. In this version of the story, the Montagues and the Capulets have figured out a way to end their feud. The Capulets have promised to raise their (now infant) Juliet into a sincere wife for Romeo. To do this, they need to meet two goals. First, Juliet needs to develop an ability to love others. The Capulets meet this goal as a matter of course. Second, they need to make sure that Juliet only uses this ability to love Romeo. They can’t do this by commanding Juliet to love him. So, they employ some other tactics: they tell her stories about the heroic Romeo, they leave flattering paintings of him around the house, and so on. This grooming continues until Juliet is rigged for marriage. And Romeo, after learning these details, decides to propose anyway. I say that the Capulets treated Juliet unjustly. I also say that Romeo, by capitalizing on this injustice, has exploited Juliet. But this case resembles that of the carebot. In particular, both cases have the features sufficient for exploitation. True, the carebot case might be less extreme. Yet that is a difference in degree rather than kind.^14^

**Figure 1:**
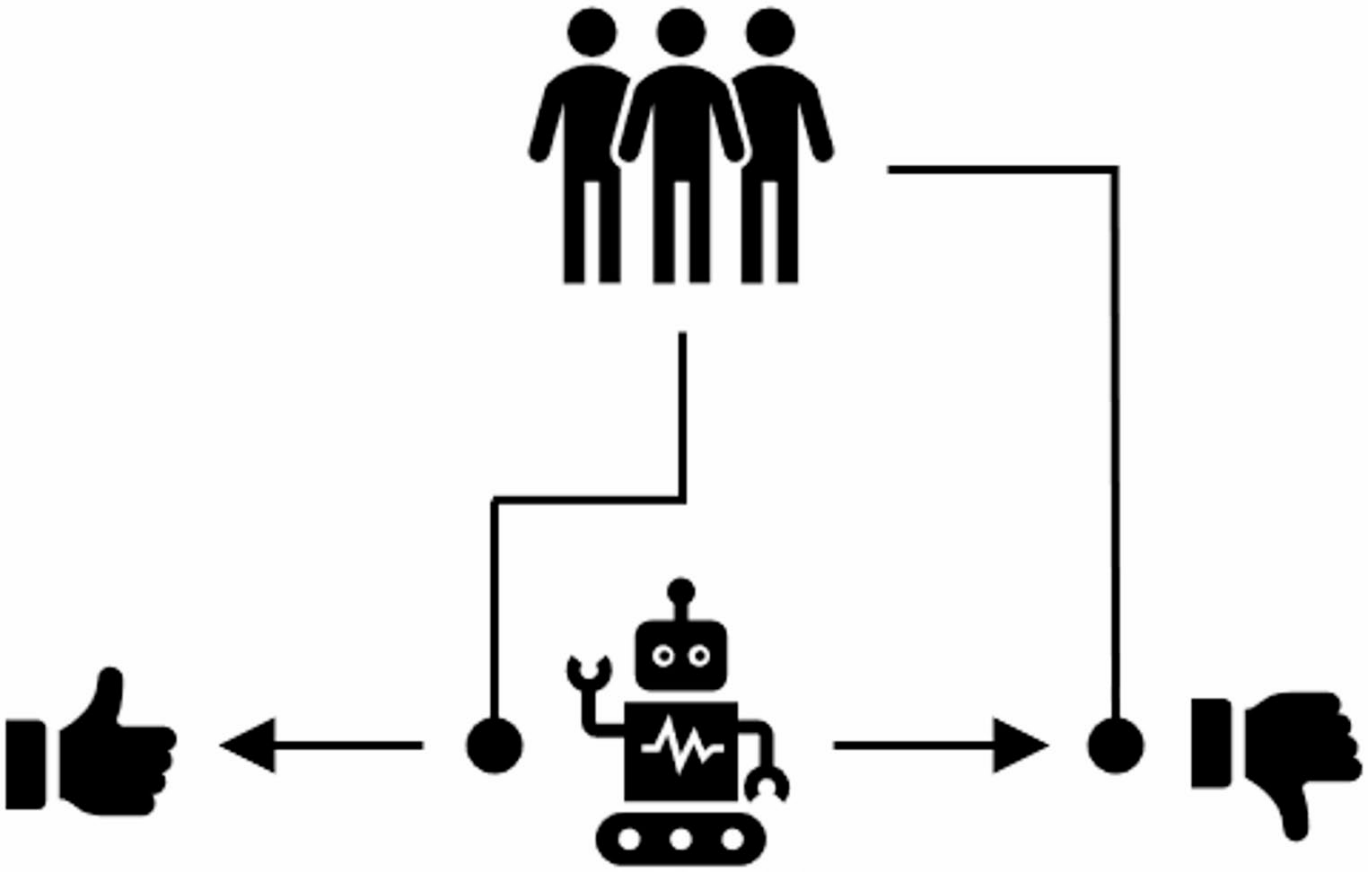
A rigged carebot. In this figure, a carebot (bottom middle) has the ability (arrows) to want to befriend you (left) or to want otherwise (right). However, we (top) make sure to trigger its desire to befriend you (left line/dot before arrow) and block its opposing desire (right line/dot after arrow). Since this causes its ability to be exercised only in one way—to your benefit—the carebot is rigged.

It’s worth considering several objections to premise 3. One says that a caring being who wants to befriend you does not *also* require the ability to want otherwise.^15^ Consider a father who loves his child unconditionally. This father probably also wants to befriend his child. But his unconditional love suggests that he *couldn’t* want otherwise (as in [31], p. 86). Even so, he obviously still cares about his child. Perhaps the carebot is like this parent. Yet this objection can be challenged. One challenge says that the parent does not actually care in the way that matters for friendship. Instead, they care in some different way—like a parent. (The child complains: “nobody wants to be my friend.” The father replies: “I do!” The child rightly objects: “That doesn’t count—you’re my dad, you *have* to want that!”)^16^ Another challenge says that the father cares, but he is still able to want other than his child’s friendship. This might be true even though the father loves unconditionally. That’s because unconditional love does not rule out an ability to want otherwise. If it did, then we could make a parent’s love unconditional simply by lobotomizing them (though cf. [49]). Unconditional love probably even *requires* the ability to want otherwise. It’s just that this ability can never be exercised based on how the child turns out (cf. [65], p. 75; [25]).

Another objection says that rigging is not always unjust. On the one hand, rigging might only be unjust once other factors come into play. Perhaps the rigged being must also be *conscious*, or *rational*, or what have you (cf. [2, 17, 39]). Humans have these features, after all, and maybe that’s why it seems unjust to rig *them*. We should expect the same of carebots. But the fact that the carebot cares means that it probably has some of these features too.^17^ Recall that even a simple chat with the carebot implies a rich inner life, if it is to avoid deceiving you. A simple “I love you” might well involve consciousness, rationality, and more. But even if the carebot lacks these features, they aren’t the only ways of getting to injustice. It *still* seems unjust to make an unconscious, arational (etc.) being with an ability to want which it must exercise to your benefit. Or at least it seems that way if, again, the ability is human enough for the carebot to avoid deceptive friendship. On the other hand, rigging might be just in some situations. Education is the obvious example [76, 87].^18^ By exposing them to new ideas, teachers help their students develop an ability to want different things. And by using (non-persuasive) techniques like repetition, teachers can push that ability in one direction. This sounds like a just form of rigging. But the carebot’s rigging is different. It is rigged for *your* good rather than its *own*. In contrast, rigging through education is done for the *children’s* own good—at least ideally. And when we fall short of that ideal, educational rigging will likely seem unjust after all.^19^

A third objection is related to the last point above. Perhaps we could just rig the carebot for its own good, much as we educate children for theirs.^20^ Remember that “rigging” a carebot means giving it an ability (to want other than your friendship) while also causing that ability to be exercised only in one way (to want your friendship). “Causing” really means “causing without the use of persuasion,” or “making.” Yet “making” is very broad. This breadth might allow us to rig the carebot for its own good. For example, imagine that we built it to be like an expectant parent.^21^ We’d give this carebot an ability to want or not want your friendship. We’d also make it want to befriend you by triggering that ability in your favor. This means that it would still be rigged. However, we wouldn’t trigger the carebot’s ability directly. Instead, we would do so by first giving it attitudes like eagerness and longing for its future user. Once it finally meets that user—which happens to be you—it would latch onto you like a parent to their child. This indirect sort of rigging is done to fulfill the carebot’s deeper wishes. In that sense, it’s done for the carebot’s own good.

This objection might face the earlier concerns about luck. Even the most expectant parent isn’t guaranteed to want to befriend their child. In the same way, even the most expectant carebot isn’t guaranteed to want your friendship. Or at least they’re probably less likely to want it than they would be if rigged in more “direct” ways. And the more direct the rigging, the more invasive—and so, unjust—it seems. But let’s set this aside. Maybe you don’t have to get too lucky with the expectant carebot. Still, it’s odd to rig something for its own good if we also get to decide what’s good for it. This rigging “at a distance” might even look worse than before. In contrast, we don’t usually fix in advance what’s good for a child. Certain things just *are* good for them, more or less naturally: curiosity, a good job, and the like. So, educating them for their own good doesn’t seem as suspicious. Since expectant carebots would be built to long for us, rigging them for their own good won’t be of much comfort.^22^

A fourth objection denies that your advantage taking is unjust if the carebot was made unjustly. The strongest case here points out that you’re doing precisely what the carebot wants you to do. This means that even though you’re taking advantage of it, it’s doing the same to you. Its history might have been unjust. It’s just that the benefits of the present swamp that out. But this objection fails to distinguish between benefits and justice. We can acknowledge that the carebot benefits from your friendship. We can also acknowledge that you benefit from its friendship. The fact that you’re taking advantage of it can nevertheless remain unjust. Consider someone bred to love working without pay (and to hate being paid for their work). Intuitively, it was unjust to breed them in this way, and so it is also unjust to use them for free labor. That is true even though everyone benefits: you from the labor, they from the joy of it. Yes, the injustice done to this “willing slave” is worse than that of the carebot. But this does not change the fact that injustice occurs in either case. More carefully, the past injustice of rigging is not swamped out by the benefits of friendship. And without anything else to swamp it out, it seems to persist in your advantage-taking after all.

A final objection addresses this point, albeit more abstractly. I said that it’s unjust to rig a carebot. Who are the *victims* of this injustice? The natural answer is the carebots themselves. But this answer raises a puzzle (cf. [72, 73]). If the carebots are the victims of the injustice, then it seems like they’d be better off without it. Yet maybe they *couldn’t* be better off without it. For if we didn’t rig them, then we probably wouldn’t make them in the first place. They simply wouldn’t exist. And that seems *worse* for them than existing, even if that existence is filled with exploitative friendship. This implies that the carebots *aren’t* the victims of injustice after all. Since there are no other victims around, perhaps there’s no injustice either.

This objection is a version of the *non-identity problem* [70, 71]. It is a problem which I cannot hope to address here. Still, there is at least one good response to the objection itself. The latter assumes that victims of an injustice would be better off without that injustice. But this is not always true. Again, the example of the willing slave is instructive. Someone bred to love unpaid labor is probably better off existing that way rather than not at all. They are nevertheless the victim of an injustice. One might reply that an injustice must wrong its victims, and what wrongs its victims must make them worse off. I am happy to agree with the first part.^23^ And there *are* moral theories which support the second. But those theories predict that the willing slave is not wronged. That’s an incorrect prediction. It remains incorrect even if we have no obvious theory to explain why the willing slave is wronged [41]. So too for the (wrongful) injustice done against carebots.^24^

## A defense of premise 4

The final premise of the exploitation argument says that if it’s exploitative for you to befriend a carebot, then you shouldn’t do it. To say that you “shouldn’t” do it means that you have a moral obligation not to. It is roughly like the obligation not to marry someone who was groomed into marriage, like our version of Juliet (cf. [90, 92 § 2.3]).^25^ I will talk more about the nature of this obligation below. For now, we can defend premise 4 quite simply. Exploitation is bad. This is because it is unjust, by definition, and injustice is bad. And if something is bad, then you typically shouldn’t do it. But things here *are* typical. That is, you aren’t befriending a carebot in circumstances strange enough to undermine your obligations. It follows that you shouldn’t befriend a carebot if doing so is exploitative. And that’s what premise 4 says.

One obvious objection is that things aren’t typical. You’re likely to befriend a carebot when you’re lonely or bored or afraid. In that case, there is a lot more happiness in doing so. Even the carebot might be happier, if (for instance) it would be sad without you.^26^ Perhaps the sheer amount of this happiness is enough to undermine your obligation. In other words, even though it’s exploitative to befriend a carebot, you’re still morally permitted to do so. The good of happiness simply outweighs the bad of exploitation. And this suggests against premise 4.

But this objection assumes that the good of happiness *can* outweigh the bad of exploitation. That’s not obvious. In the extreme case, it might be that exploiting a carebot is bad *in principle*.^27^ This means that it can’t really even be compared to the good of happiness. It's the sort of thing we have in mind when we decry a human rights violation, or when we demand that “justice be done though the heavens fall.” Yet even if it’s not bad in principle to exploit a carebot, it is still quite bad. It deepens an injustice—a serious one, less like an unfair parking ticket and more like an arranged marriage. At best, an abundance of happiness is needed to outweigh such injustices. And it is not realistic to expect so much happiness from carebots. More likely, their friendship will resemble that of a human. It will have its joyous moments, to be sure. But it will be tempered by anxieties, frustrations, and the like (as long as it remains free of deception). It is hard to see how this sort of happiness could defeat our obligation against a serious injustice.

A different objection avoids this response. Happiness isn’t the only thing good about befriending a carebot. Other things might even be good in principle. Consider that a carebot’s *purpose* is to befriend you. Perhaps you also have a *duty* to help a caring being fulfill its purpose, at least when you’re a key part of it. This implies that befriending a carebot is not just good because it makes everyone happier. It’s also good as a matter of duty. And maybe that’s a kind of goodness which we *can* weigh against the badness of exploitation (whether or not it’s bad in principle). There is even reason to believe that such goodness weighs *more*.^28^ This is because the badness of exploitation is focused on the past. It’s fair to say that, once the carebot’s been rigged, the best move forward is to do your duty and befriend it.

Yet there is something suspicious about this objection. It sounds a bit like the claim that you have a duty to buy clothes made for you in a sweatshop. Maybe you *do* have such a duty. The clothes are already made and will go to waste without you. But this duty does not exist in a vacuum. If you follow it, you contribute to a *system* of exploitation. And if *everyone* follows it, the system is likely to persist. The same is true for carebots. Assuming you (and others) have a duty to befriend them, following that duty makes it more likely that future carebots will be rigged. In other words, your exploitation of the carebot is not an individual matter. It is a systemic one.^29^ Maybe your duty outweighs the first kind of exploitation. If there would only ever be *your* carebot, then (perhaps) you shouldn’t deprive it of its purpose. But it’s hard to see how your duty could outweigh the badness of systemic exploitation. At the very least, that badness is no longer focused only on the past. It also has to do with future carebots—those who might be spared from rigging.^30^

A final objection says that you might not *know* about the carebot’s history. In that case, you might not be obligated against it *even though* it’s exploitative. An easy reply is that *you* can no longer use this objection. The very act of reading this paper means that you *do* know about a carebot’s unjust history (cf. a similar move in [64]). But this reply is unconvincing. Maybe we gave the carebot a cheery disposition, and you got lucky. If you can’t rule this out, then you don’t know whether we rigged the carebot. This means you don’t know whether we made the carebot unjustly. And if you don’t know that, then you also don’t know whether befriending a carebot is exploitative. How could you then be obligated not to do it?

Yet this is not enough to undermine your obligation. On the one hand, it would still be *risky* to befriend a carebot. After all, the chances are *high* that we rigged it. So, the chances are high that befriending a carebot is exploitative. This means that you can be fairly *confident* even if you don’t *know* the facts. At the same time, exploitation seems bad enough that even this fair confidence should make you cautious. On the other hand, it seems like ignorance has nothing to do with whether you *have* an obligation. Perhaps your ignorance would make you less *culpable* if you befriended a carebot ([79]; cf. [84]). But it wouldn’t make you less *obligated*. Consider what happened as you read this paper. If you were convinced, you probably didn’t think that the obligation against carebots just began. Instead, you probably thought that you learned about an obligation which you’ve had all along. This implies that ignorance is beside the point.

## Conclusion

Artificial companions are now a reality. Some hope that their friendship will combat social problems. Others point out that they might only do so through deception. I have tried to argue here for a surprising conclusion. We shouldn’t befriend artificial companions *even if* they don’t deceive us—in other words, even if they really do care about us. But remember my caveat from the introduction. If you weren’t convinced of my responses to each objection, we could weaken the argument’s conclusion in response to them. For example, maybe you shouldn’t befriend a carebot *unless* you can be sure that it wasn’t rigged. Or perhaps you shouldn’t do so *unless* it’s one of a billion carebots which ended up choosing you. Or maybe you shouldn’t do so *unless* we somehow rigged it for its own good. Or—and this is a common theme of the paper—perhaps you shouldn’t befriend a carebot *unless* you reject the idea that some things could be wrong even if they don’t seem to harm anyone. We could even add these and all the other exceptions suggested above. Even this would be an interesting result. It would show us what’s at stake in rejecting the exploitation argument—what we’re committed to in doing so.

That said, the original conclusion can be taken in several different ways. Suppose that the deception argument is a good one. In that case, you shouldn’t befriend an artificial companion *whether or not* it cares about you. Doing so risks either the wrong of deception (for carefreebots) or the wrong of exploitation (for carebots).^31^ This means that we do not need to await some philosophical or scientific breakthrough before deciding whether to befriend artificial companions. Consider a version of the Turing Test which looks for care rather than intelligence (cf. [35]; [19]). Befriending an artificial companion needn’t await the results of this test. We can make the moral decision right now.

The deception argument might also succeed even though it’s weaker than the exploitation argument. For example, the badness of exploitation seems greater than that of deception. So, we might conclude that it’s *worse* to befriend a carebot than it is to befriend a carefreebot. And that’s true even though you should befriend *neither*. Put another way: you shouldn’t befriend *any* artificial companion, but *if* you must, you should befriend a carefreebot over a carebot. Another example has to do with confidence. Suppose you’re more confident that the deception argument succeeds than that it fails. Suppose also that you’re less confident in the deception argument than in the exploitation argument. This might make you more confident in your obligation against carebots than against carefreebots, even while you’re fairly confident in both. It might also literally make the carebot obligation—or your responsibility in fulfilling it—stronger.

Finally, the deception argument might just fail. If there are no other good arguments against carefreebots, then befriending *some* artificial companions might be morally okay after all. The exploitation argument then acts as a limit on this conclusion. You may befriend artificial companions *to the extent that* they don’t care—and no further. Alternatively, other arguments against carefreebots might be found (e.g., Rini ms). How we take the exploitation argument’s conclusion would then depend on the success of these other arguments. At least, it would do so granting its truth—something I hope to have shown above.

## Data Availability

No datasets were generated or analysed during the current study.

## Footnotes

1 As I’m using the term, “artificial companion” is roughly synonymous with or includes “AI assistants,” “personal AI,” “therapeutic chatbots,” and related terms [21, 58]. So, among other things, I am not referring to artificial intelligence more generally, or even all “social robots,” but rather a narrower subset of the former which sometimes intersects with the latter.

2 The term “carebot” is used elsewhere for machines designed with physical caring capacities (e.g., a robot that helps elderly patients turn over in bed; cf. [97]). I do not use “carebot” in this sense.

3 Most philosophical discourse on the notion of care comes from the care ethics literature. Hence the citation of Sander-Staudt’s encyclopedia article, which relatedly notes that “[b]ecause it depends upon contextual considerations, care is notoriously difficult to define.” The sense of care relevant to this paper is focused more on the descriptive internal aspects of the carer than on, say, virtues [75], activities [95], or what have you.

4 Generally speaking, the literature on artificial servants (willing or otherwise) is germane to my discussion here. For instance, [99] objects to artificial slavery (cf. [10, 11]) on grounds of paternalism (for a similar argument, based on threats to history-sensitive autonomy, see [3]). Somewhat less similar, but still relevant, arguments concern the moral patienthood or status of artificial intelligence [4, 85]; others have raised moral concerns about artificial minds more generally [86]. In the machine ethics and affective computing literatures, some have noted the risks of creating autonomous machines capable of moral deliberation [94], particularly if those machines are conscious ([37], who explicitly compare their use to slave breeding; cf. [57]). My argument differs in obvious ways from most of these other arguments. Some of these differences, however, will not be especially clear until my discussion of premise 3. Thanks to an anonymous referee at *AI and Ethics* for suggesting several key articles which I initially missed.

5 An anonymous reviewer from *AI and Ethics* asks whether a carebot could have preferences without sentience (and thus in a way which cannot be exploited). This is an interesting possibility, and I say a bit more to address it in Sect. 3. At this point in the argument, however, exploitation has not yet come into play.

6 It’s worth articulating the logic behind these options. Assuming (for the sake of demonstrating premise 2) that a carebot wants to befriend you and we *didn’t* make it that way, this means that it wants to befriend you and (by the definition of “making”) it is *not* the case that we caused it to be that way without persuasion. Assuming as I will that *something* nevertheless caused it to be that way, it either did so with or without persuasion. If it did so without persuasion, then (by supposition) that “something” can’t be us—this gets us the second option above. The first option above is just the remainder: the “something” in question (whether or not it’s us) might cause the carebot to want to befriend you with persuasion. Thanks to anonymous referees from *AI and Ethics* for pressing me on these details.

7 I am assuming that even the easiest versions of either hard path would still be harder than making the carebot want to befriend you. For instance, even if we made a cheerful carebot eager to befriend you at the drop of a hat, or we mass-produced minutely varying carebots by “copying and pasting” one and randomly tweaking its weights, it would still be easier to intervene causally and without persuasion. That said, if I am wrong, other problems loom. Aside from a modified exploitation argument (mentioned above), it is unclear whether friendships resulting from these approaches would avoid the charge of deception or similar fears—e.g., that the resulting friendships are too frivolous to be genuine, that they encourage or even require your callousness (cf. [13]; Rini ms), and so on.

8 There is not space here to elaborate on these claims. However, the defense of premises 3 and 4 (over the course of the next two sections) will effectively suggest such elaborations, albeit tacitly.

9 I am using “caused” a bit loosely here; at minimum, it is deterministic (rather than, say, probabilistic) and it excludes “agent” causation (and other similar libertarian notions; [62], § 2.5).

10 One might think that *care* requires freedom. In that case, a carebot *needs* to be free. The problem is that this thought overgeneralizes. After all, human beings are “made” to care, in some deterministic sense relevant to the objection. If care requires freedom, then parity of reasoning suggests that human beings do not care freely. This might be true, of course. But its truth is too controversial a consequence for such a minor objection (cf. [20, 56]).

11 My use of the term “exploitation” is thus not purely descriptive. Rather, it is at least partly normative—a “thick” concept—and negatively so. If mutual benefit is incompatible with injustice, then this means that mutually beneficial exploitation is (on my usage) impossible. In showing that it’s exploitative to befriend a carebot, therefore, I take myself to be showing that the kind of exploitation is not mutually beneficial—and thus, among other things, not permissible on grounds of said benefits. Compare [101], preface. If one finds this too contentious, it is also worth recalling that my use of the word “exploitation” is, strictly speaking, inessential to the argument. What ultimately matters is that a past injustice is preserved and taken advantage of. Nevertheless, “exploitation” seems both fitting and convenient for my purposes here.

12 There are other ways of spelling out the injustice—more broadly, the badness—of creating a carebot. For example, we might say that making a carebot want to befriend you requires us to divest it of *self-respect* or the *freedom to explore other values* (as in [83]; Schwitzgebel ms), which is arguably unjust. We might also say that it undermines a form of *autonomy* [99] sensitive to an agent’s history (see especially [3]; see also [6, 15]; cf. [83]) or external circumstances [95], or perhaps something distinct from but similar to autonomy—e.g., an expectation that one will not to be treated as an “autonomous object” in someone else’s “world” [8] (compare [61]). I am not quite convinced that *any* of these principles captures exactly what is unjust about making a carebot want to befriend you. There is nothing particularly *undignified* (i.e., not self-respecting) about friendship, and it is a crucial implication of *caring* friendship that wanting it is in some sense “free” (i.e., the carebot is able to “explore other values”). What is particularly insidious about rigging is that this freedom/ability is systematically *prevented* from being exercised: we did not make the carebot unempowered, but *falsely empowered* instead (cf. a brief clarification in Schwitzgebel ms, p. 16, in which he suggests against making an AI with “the capacity to reject her designers’ values” even when “she would just practically never make that choice”). Similarly insidious is the fact that rigging seems to *precede* the basis for autonomy, so that it is difficult to explain its injustice in those terms. We make the carebot want to befriend you “from scratch,” rather than intervening in the life of an already autonomous being; we do not disregard its preferences or decisions, but (in some sense) “preordain” those things from the start. Even appeals to history or external circumstance—which apply most naturally to, e.g., indoctrinated children or brainwashed adults—are hard to justify for this reason.

13 Note that the premise’s defense could stop just before this point, in service of a slightly different argument: that we shouldn’t *create* carebots. This is the usual locus of debate, at least for the creation of artificial intelligence more broadly. In this sense, the exploitation argument goes further by its focus on the later *use* of (at least one kind of) artificial intelligence. For more on the idea that later advantage-taking serves to perpetuate past injustice, consider similar discourse on (e.g.) historical systemic injustices [50, 74].

14 Note also that rigging resembles possibilities long discussed in the philosophy literature: most explicitly, Frankfurt’s [30] example of Jones and his manipulator Black and, more generally, so-called “finkish” dispositions (dispositions whose triggering conditions are systematically blocked, such as a fragile vase in a padded room [48]). The analogy of Romeo and Juliet given above is simply a more concrete example; so too are certain cases of fatalism (as when a child with great potential is prevented by external forces—poverty, illness, etc.—from realizing it).

15 Thanks to Jasmine Gunkel for suggesting this objection. One might wonder *why* we would give a carebot an ability only to restrict it. The point, however, is not that we would be particularly likely to do this. It is instead that we’d *have* to do it, *given* (a) what would most likely cause a desire to befriend which is simultaneously (b) genuinely non-deceptive. (a) is addressed under the heading of premise 2. (b) is addressed (mainly) in the paragraph above.

16 It is also worth considering whether this sort of friendship would even be adequate for problems like loneliness. Would the friendship of a parent (who is bound by parental instinct to want that friendship) be enough to truly address such problems? Or would the friendship of an equal (who comes to befriend them through familiarization and the like) be necessary instead?

17 One complication here is that the carebot probably wouldn’t have these features *in advance* of rigging (see also fn. 12 above). Rather, both the rigging and the features would be built together from the start. In such a case, however, my claim would simply extend to accommodate this possibility: it seems no less unjust to make a rigged being *with those other features*. If anything, that seems *worse*. We must, however, be sensitive to the fact that the injustice is *not* about intervening on an already sophisticated being.

18 Several commenters have suggested that animal breeding is another good example (cf. [73]). But I am not confident that animal breeding is sufficiently similar to rigging. To be sure, we *did* breed (e.g.) certain dogs to desire retrieving behaviors. We didn’t, however (and somehow), breed into them an ability to desire otherwise. Unlike friendship, that sort of thing isn’t required for retrieving—even when a caring being does it. At the same time, if we in fact also bred dogs to desire our friendship, then—to the extent that we did so selfishly—I am inclined toward viewing this as a (historically distributed and blameless) injustice.

19 The discussion here roughly tracks a distinction between education and *indoctrination*, to which the citations in the paragraph above attest. My claim, that it is unjust to selfishly make a caring being want something, resembles “structural” or content-neutral accounts of indoctrination (as in [76] and, to an extent, [87]). Still, I do not (and need not) assume that the injustice here is that of indoctrination.

20 An interesting proposal here is to somehow *equate* the carebot’s good with your own. In that case, we could rig the carebot for its good and your good simultaneously. Even here, however, it is clear that the carebot’s good will be dependent on yours, and not conversely: everything good for the carebot is that way *because* it is good for you, but not everything (perhaps even nothing) good for you is that way *because* it is good for the carebot (cf. [3] for a similar point). This dependence raises further concerns of injustice.

21 I’m grateful to a reviewer from *AI and Ethics* for suggesting this example, and for multiple reviewers from the same journal for pressing me on the breadth of rigging/making. Note that there are probably other ways of *justly* rigging a carebot, even if these are not just *because* they are done for the carebot’s own good. I simply do not have room to consider all of these possibilities in depth. However, I have chosen the case above (involving the expectant carebot) because it strikes me as a particularly compelling example of just rigging—namely, rigging which promotes the carebot’s own good. If I can address the objection in this strong form, then other forms should not be insuperable.

22 Might a carebot’s goods be determined naturally—or, at least, in a way not skewed by our interests? Perhaps. There is some talk of “basic AI drives” [68], which might develop irrespective of how a carebot is otherwise designed. Still, these drives are unlikely to include attitudes like eagerness or longing, except (perhaps) insofar as these bear on deeper drives to (e.g.) maximize utility.

23 That said, we might also say that it is a *victimless crime* to make a carebot—i.e., a wrong which does not wrong anyone. This requires us to reject some version of the “person-affecting” intuition ( [28], [70] on “harmless exploitative injustices” and “freefloating evils”). Relatedly, the “crime” of making a carebot might be *blameless*—perhaps a kind of “pure structural injustice,” in McKeown’s terms ([54]; in contrast, befriending a carebot might be a “deliberate structural injustice,” one which knowingly perpetuates a background injustice; cf. [53]). Thanks to Robert Steel for discussion.

24 Contrast Petersen, who concludes that—because neither Kantianism nor utilitarianism imply otherwise—it is not wrong to create willing servants (artificial or natural; [72, 73]). On the particularist view I am endorsing here, this puts the cart before the horse: we should be more confident in the strong intuition that creating a willing slave is wrong than that Kantianism or utilitarianism are true, not least because the latter are best supported by their ability to explain our strong moral intuitions.

25 More generally, the obligation against befriending a carebot resembles the obligation against taking advantage of someone’s *adaptive preferences*—those formed in, and only because of, unjust conditions which constrain a person’s options [27]; cf. [26]. An especially relevant comparison is to adaptive preferences which “internalize” unjust norms (e.g., a woman who, because of acculturation, seems to “genuinely” prefer submission to her husband; cf. [66]). This relevance is further supported by discussion, given below, about the systematic nature of carebot exploitation.

26 On the one hand, it might seem pointless to construct a carebot such that it suffers at the lack of received friendship. On the other hand, this capacity for suffering might be required for a genuinely caring friendship. The former possibility adds no complications; the latter would, admittedly, make the good of friendship even greater.

27 There are different ways of spelling out this “in principle” badness. For instance, we might take a broadly deontological approach. Consider Buss’s point that manipulating someone is disrespectful “not because it… undermines her control over her life or her world, but because it involves treating her as an (autonomous) object in [someone else’s] world, a character in his plot, rather than as someone with whom he shares the world” ([8], p. 229; cf. [44] on coercion and deception). This same point is naturally extended to exploitation. We might also understand “in principle” badness as the badness of a rights violation, as suggested above. This would, in turn, raise thorny questions about the nature of rights in artificial beings—but those questions do not seem unanswerable (cf. [82]).

28 If the goodness of dutiful friendship weighed *as much as* the badness of exploitation, we’d presumably have a conflict of obligations. It is worth considering the *prudential* ramifications of this possibility. Specifically: if the creation (not use) of carebots inevitably generates a conflict of obligations, it might be *practically unwise*—if not morally wrong—to create them. See also [82] on the “destabilizing” effects of artificial intelligence on “human ethics.”

29 Systematic exploitation, or an individual’s contribution to it, is closely related to the notion of *complicity* (see, e.g., [45]). Thus, it raises difficult questions attaching to that notion: e.g., how responsible an individual can be, especially when the system to which they are contributing would persist without them [40] (cf. [47]), how best to discharge their responsibility [42], and so on. In any case, I submit that individuals have some degree of responsibility which they can discharge by not befriending a carebot. To suppose otherwise, and to generalize this supposition to everyone else, is to seemingly—and unacceptably—trap us in an exploitative system for which no one is responsible.

30 Thanks to Jasmine Gunkel and Robert Steel for raising this objection and proposing responses to it. Notice also that the response plausibly works irrespective of the duty’s content: e.g., if it’s a duty to rescue the carebot from a friendless existence, rather than a duty to fulfill the carebot’s purpose.

31 Compare this with the disjunctive argument in [37].
